# 1-Benzyl-6-phenyl­imino-5-(pyrrol-2-yl­idene)hexa­hydro­pyrimidine-2,4-dione

**DOI:** 10.1107/S1600536808001384

**Published:** 2008-01-23

**Authors:** Rafael Tamazyan, Armen Ayvazyan, Vahan Martirosyan, Kristine Avagyan, Ashot Martirosyan

**Affiliations:** a Molecule Structure Research Center, National Academy of Sciences RA, Azatutyan Avenue 26, 375014 Yerevan, Republic of Armenia; b Institute of Fine Organic Chemistry, National Academy of Sciences RA, Azatutyan Avenue 26, 375014 Yerevan, Republic of Armenia

## Abstract

In the title compound, C_21_H_20_N_4_O_2_, a potential anti-human immunodeficiency virus type 1 (HIV-1) non-nucleoside reverse transcriptase inhibitor, the pyrrolidine ring adopts an envelope conformation, while the hydrogenated pyrimidine ring adopts a weakly expressed twist conformation. The mol­ecules are connected into infinite chains *via* N—H⋯O hydrogen bonds.

## Related literature

For related structures, see: Karapetyan *et al.* (2002 ▶); Tamazyan *et al.* (2002 ▶). For details of the pharmacological properties of similar compounds, see: De Clercq (1996 ▶).
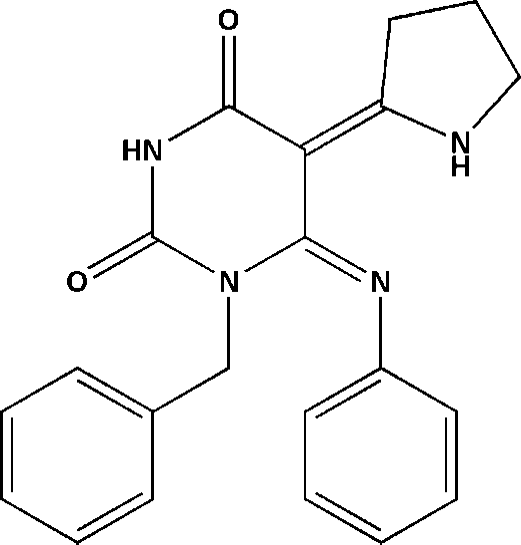


## Experimental

### 

#### Crystal data

C_21_H_20_N_4_O_2_
*M* *_r_* = 360.41
Triclinic, 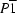

*a* = 5.7844 (12) Å
*b* = 10.378 (2) Å
*c* = 15.595 (3) Å
α = 102.64 (3)°
β = 93.32 (3)°
γ = 102.45 (3)°
*V* = 886.6 (3) Å^3^
*Z* = 2
Mo *K*α radiation
μ = 0.09 mm^−1^
*T* = 293 (2) K
0.3 × 0.27 × 0.25 mm

#### Data collection

Enraf–Nonius CAD-4 diffractometer
Absorption correction: none
5619 measured reflections
5144 independent reflections
2944 reflections with *I* > 2σ(*I*)
*R*_int_ = 0.028
3 standard reflections frequency: 180 min intensity decay: none

#### Refinement

*R*[*F* ^2^ > 2σ(*F* ^2^)] = 0.065
*wR*(*F* ^2^) = 0.128
*S* = 1.04
5144 reflections
325 parameters
All H-atom parameters refined
Δρ_max_ = 0.27 e Å^−3^
Δρ_min_ = −0.26 e Å^−3^

### 

Data collection: *CAD-4 Software* (Enraf–Nonius 1988 ▶); cell refinement: *CAD-4 Software*; data reduction: *HELENA* (Spek, 1997 ▶); program(s) used to solve structure: *SHELXS97* (Sheldrick, 2008 ▶); program(s) used to refine structure: *SHELXL97* (Sheldrick, 2008 ▶); molecular graphics: *SHELXTL* (Sheldrick, 2008 ▶) and *ORTEPII* (Johnson, 1976 ▶); software used to prepare material for publication: *SHELXTL*.

## Supplementary Material

Crystal structure: contains datablocks global, I. DOI: 10.1107/S1600536808001384/pv2060sup1.cif

Structure factors: contains datablocks I. DOI: 10.1107/S1600536808001384/pv2060Isup2.hkl

Additional supplementary materials:  crystallographic information; 3D view; checkCIF report

## Figures and Tables

**Table 1:** Hydrogen-bond geometry (Å, °)

*D*—H⋯*A*	*D*—H	H⋯*A*	*D*⋯*A*	*D*—H⋯*A*
N10—H10⋯O7	0.90 (2)	2.03 (2)	2.664 (3)	127 (2)
N10—H10⋯O7^i^	0.90 (2)	2.22 (2)	2.891 (3)	131 (2)
N3—H3⋯O8^ii^	0.86 (2)	2.10 (2)	2.937 (3)	165 (2)

Symmetry codes: (i) 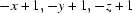; (ii) 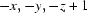.

**Fig. 1.**
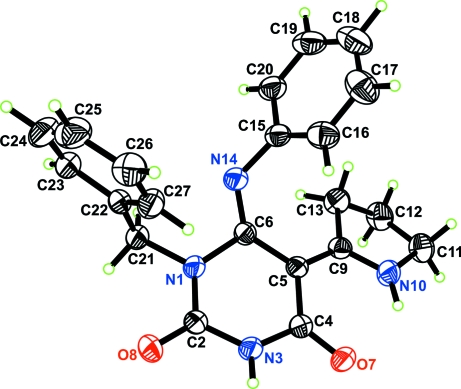
A view of (I) with the atomic numbering scheme. Displacement ellipsoids are drawn at the 50% probability level.

**Fig. 2.**
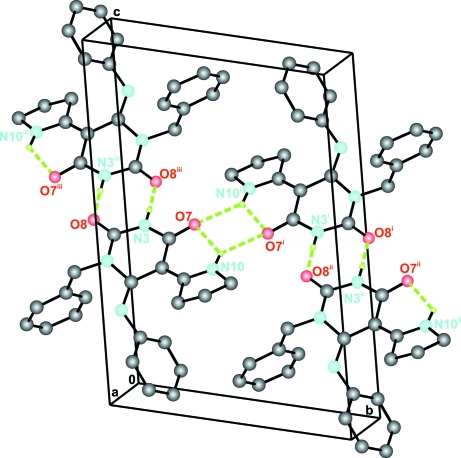
Unit cell packing of (I) showing infinite chains of molecules via hydrogen bonding; for clarifity only H atoms participating in bonding have been included. Symmetry cods: (i) 1 - x, 1 - y, 1 - z; (ii) 1 + x, 1 + y, z; (iii) -x, -y, 1 - z.

## supplementary crystallographic information

### Comment

The interest to X-ray structural investigation of the title compound, (I), was stimulated by its potentially HIV-1 RT inhibition properties. It belong to the family of non-nucleoside reverse transcriptase inhibitors (NNRTIs).

A view of (I) with our numbering scheme is depicted in Fig. 1. A l l intramolecular interatomic distances in (I) are in good agreement with their mean statistical values. The crystal structure consists of infinite chains along [112] direction of crystal lattice. These chains are formed by molecules of (I) *via* O7···H10—N10 and O8···H3—N3 hydrogen bonds (Fig. 2).

### Experimental

The title compound was synthesized by the condensation of 6-anilino-1-benzyl-1,2,3,4-tetrahydro-2,4-pyrimidinedione with pyrrolidon-2. The crystals were grown from an ethanol solution. A suitable crystal of the size ~0.3 mm was selected for X-ray diffraction experiment.

### Refinement

The positional parameters of all atoms, anisotropic displacement parameters of nonhydrogen atoms and isotropic thermal parameters of hydrogen atoms were refined without restraints.

### Crystal data

**Table:**

C_21_H_20_N_4_O_2_	*Z* = 2
*M**_r_* = 360.41	*F*_000_ = 380
Triclinic, *P*1	*D*_x_ = 1.350 Mg m^−^^3^
Hall symbol: -P 1	Mo *K*α radiation λ = 0.71073 Å
*a* = 5.7844 (12) Å	Cell parameters from 22 reflections
*b* = 10.378 (2) Å	θ = 13–16º
*c* = 15.595 (3) Å	µ = 0.09 mm^−^^1^
α = 102.64 (3)º	*T* = 293 (2) K
β = 93.32 (3)º	Prism, colourless
γ = 102.45 (3)º	0.3 × 0.27 × 0.25 mm
*V* = 886.6 (3) Å^3^	

### Data collection

**Table:**

Enraf–Nonius CAD-4 diffractometer	*R*_int_ = 0.028
Radiation source: fine-focus sealed tube	θ_max_ = 30.0º
Monochromator: graphite	θ_min_ = 1.4º
*T* = 293(2) K	*h* = 0→8
θ/2θ scans	*k* = −14→14
Absorption correction: none	*l* = −21→21
5619 measured reflections	3 standard reflections
5144 independent reflections	every 180 min
2944 reflections with *I* > 2σ(*I*)	intensity decay: none

### Refinement

**Table:**

Refinement on *F*^2^	Hydrogen site location: difference Fourier map
Least-squares matrix: full	All H-atom parameters refined
*R*[*F*^2^ > 2σ(*F*^2^)] = 0.065	*w* = 1/[σ^2^(*F*_o_^2^) + (0.0198*P*)^2^ + 0.561*P*] where *P* = (*F*_o_^2^ + 2*F*_c_^2^)/3
*wR*(*F*^2^) = 0.128	(Δ/σ)_max_ < 0.001
*S* = 1.04	Δρ_max_ = 0.27 e Å^−^^3^
5144 reflections	Δρ_min_ = −0.26 e Å^−^^3^
325 parameters	Extinction correction: SHELXL97 (Sheldrick, 2008), Fc^*^=kFc[1+0.001xFc^2^λ^3^/sin(2θ)]^-1/4^
Primary atom site location: structure-invariant direct methods	Extinction coefficient: 0.0124 (13)
Secondary atom site location: difference Fourier map	

### Special details

**Table:**

Geometry. All e.s.d.'s (except the e.s.d. in the dihedral angle between two l.s. planes) are estimated using the full covariance matrix. The cell e.s.d.'s are taken into account individually in the estimation of e.s.d.'s in distances, angles and torsion angles; correlations between e.s.d.'s in cell parameters are only used when they are defined by crystal symmetry. An approximate (isotropic) treatment of cell e.s.d.'s is used for estimating e.s.d.'s involving l.s. planes.
Refinement. Refinement of *F*^2^ against ALL reflections. The weighted *R*-factor *wR* and goodness of fit *S* are based on *F*^2^, conventional *R*-factors *R* are based on *F*, with *F* set to zero for negative *F*^2^. The threshold expression of *F*^2^ > σ(*F*^2^) is used only for calculating *R*-factors(gt) *etc*. and is not relevant to the choice of reflections for refinement. *R*-factors based on *F*^2^ are statistically about twice as large as those based on *F*, and *R*- factors based on ALL data will be even larger.

### Fractional atomic coordinates and isotropic or equivalent isotropic displacement parameters (Å^2^)

**Table:**

	*x*	*y*	*z*	*U*_iso_*/*U*_eq_	
N1	0.4204 (3)	−0.03850 (16)	0.35608 (11)	0.0320 (4)	
C2	0.2737 (4)	−0.0209 (2)	0.42132 (14)	0.0329 (5)	
H3	0.163 (4)	0.120 (2)	0.4942 (16)	0.043 (7)*	
N3	0.2672 (4)	0.11039 (18)	0.45813 (13)	0.0374 (5)	
C4	0.4114 (4)	0.2259 (2)	0.44199 (13)	0.0312 (5)	
C5	0.5484 (4)	0.20422 (19)	0.36835 (13)	0.0291 (4)	
C6	0.5188 (4)	0.0671 (2)	0.31427 (14)	0.0295 (4)	
O7	0.4116 (3)	0.33653 (15)	0.49159 (10)	0.0430 (4)	
O8	0.1540 (3)	−0.11624 (15)	0.44595 (11)	0.0469 (4)	
C9	0.7255 (4)	0.3145 (2)	0.35785 (13)	0.0300 (4)	
H10	0.638 (4)	0.464 (2)	0.4363 (16)	0.044 (7)*	
N10	0.7427 (4)	0.44206 (18)	0.39838 (13)	0.0393 (5)	
C11	0.9511 (5)	0.5384 (2)	0.3816 (2)	0.0465 (6)	
H11A	1.017 (5)	0.610 (3)	0.438 (2)	0.075 (9)*	
H11B	0.906 (5)	0.585 (3)	0.3357 (18)	0.060 (8)*	
C12	1.1099 (5)	0.4461 (3)	0.34836 (19)	0.0462 (6)	
H12A	1.208 (5)	0.435 (3)	0.4041 (19)	0.066 (9)*	
H12B	1.215 (5)	0.476 (3)	0.3058 (17)	0.058 (8)*	
C13	0.9361 (4)	0.3101 (3)	0.30583 (17)	0.0401 (6)	
H13A	0.993 (5)	0.228 (3)	0.3025 (17)	0.054 (8)*	
H13B	0.890 (5)	0.310 (3)	0.245 (2)	0.071 (9)*	
N14	0.5687 (3)	0.02327 (18)	0.23576 (12)	0.0367 (4)	
C15	0.5951 (4)	0.0974 (2)	0.16998 (14)	0.0342 (5)	
C16	0.4413 (5)	0.1784 (3)	0.15504 (17)	0.0457 (6)	
H16	0.325 (4)	0.192 (2)	0.1957 (16)	0.048 (7)*	
C17	0.4605 (6)	0.2394 (3)	0.08445 (19)	0.0578 (8)	
H17	0.351 (5)	0.292 (3)	0.0734 (19)	0.069 (9)*	
C18	0.6315 (6)	0.2211 (3)	0.02769 (18)	0.0593 (8)	
H18	0.646 (5)	0.263 (3)	−0.024 (2)	0.072 (9)*	
C19	0.7829 (5)	0.1407 (3)	0.04185 (17)	0.0533 (7)	
H19	0.904 (5)	0.127 (3)	0.002 (2)	0.072 (9)*	
C20	0.7642 (5)	0.0783 (3)	0.11150 (16)	0.0439 (6)	
H20	0.866 (5)	0.023 (3)	0.1218 (17)	0.056 (8)*	
C21	0.4193 (4)	−0.1793 (2)	0.31229 (15)	0.0350 (5)	
H21A	0.575 (4)	−0.179 (2)	0.2917 (14)	0.033 (6)*	
H21B	0.403 (4)	−0.229 (2)	0.3603 (15)	0.037 (6)*	
C22	0.2243 (4)	−0.2461 (2)	0.23746 (14)	0.0334 (5)	
C23	0.2296 (5)	−0.3733 (2)	0.18596 (16)	0.0417 (6)	
H23	0.367 (5)	−0.412 (3)	0.1979 (17)	0.058 (8)*	
C24	0.0439 (5)	−0.4443 (3)	0.12153 (18)	0.0528 (7)	
H24	0.049 (5)	−0.533 (3)	0.0869 (17)	0.059 (8)*	
C25	−0.1440 (5)	−0.3884 (3)	0.10493 (18)	0.0546 (7)	
H25	−0.276 (5)	−0.439 (3)	0.0577 (18)	0.064 (8)*	
C26	−0.1472 (5)	−0.2606 (3)	0.15307 (17)	0.0482 (6)	
H26	−0.285 (5)	−0.219 (3)	0.1429 (17)	0.061 (8)*	
C27	0.0355 (5)	−0.1909 (2)	0.21906 (16)	0.0415 (6)	
H27	0.030 (4)	−0.103 (2)	0.2539 (15)	0.038 (6)*	

### Atomic displacement parameters (Å^2^)

**Table:**

	*U*^11^	*U*^22^	*U*^33^	*U*^12^	*U*^13^	*U*^23^
N1	0.0374 (10)	0.0241 (8)	0.0323 (9)	0.0033 (7)	0.0113 (8)	0.0042 (7)
C2	0.0368 (12)	0.0286 (10)	0.0328 (11)	0.0050 (9)	0.0096 (9)	0.0071 (9)
N3	0.0432 (11)	0.0282 (9)	0.0404 (11)	0.0049 (8)	0.0219 (9)	0.0063 (8)
C4	0.0339 (12)	0.0288 (10)	0.0307 (11)	0.0062 (9)	0.0062 (9)	0.0066 (9)
C5	0.0318 (11)	0.0260 (10)	0.0284 (10)	0.0030 (8)	0.0086 (9)	0.0062 (8)
C6	0.0288 (11)	0.0259 (10)	0.0330 (11)	0.0030 (8)	0.0090 (9)	0.0072 (8)
O7	0.0566 (11)	0.0281 (8)	0.0424 (9)	0.0071 (7)	0.0204 (8)	0.0032 (7)
O8	0.0581 (11)	0.0319 (8)	0.0532 (10)	0.0052 (8)	0.0276 (9)	0.0153 (7)
C9	0.0301 (11)	0.0318 (11)	0.0268 (10)	0.0046 (9)	0.0026 (8)	0.0069 (8)
N10	0.0418 (12)	0.0279 (9)	0.0462 (12)	0.0024 (8)	0.0149 (10)	0.0079 (8)
C11	0.0464 (15)	0.0323 (12)	0.0540 (16)	−0.0060 (11)	0.0077 (13)	0.0106 (12)
C12	0.0374 (14)	0.0424 (14)	0.0541 (16)	−0.0033 (11)	0.0071 (12)	0.0136 (12)
C13	0.0340 (13)	0.0371 (13)	0.0453 (14)	0.0011 (10)	0.0120 (11)	0.0067 (11)
N14	0.0441 (11)	0.0309 (9)	0.0336 (10)	0.0047 (8)	0.0141 (8)	0.0063 (8)
C15	0.0361 (12)	0.0310 (11)	0.0289 (11)	−0.0022 (9)	0.0075 (9)	0.0022 (9)
C16	0.0410 (14)	0.0533 (15)	0.0411 (14)	0.0068 (12)	0.0079 (12)	0.0111 (11)
C17	0.0546 (18)	0.0641 (18)	0.0554 (17)	0.0070 (15)	−0.0062 (14)	0.0257 (15)
C18	0.066 (2)	0.0638 (18)	0.0395 (15)	−0.0107 (15)	−0.0006 (14)	0.0215 (13)
C19	0.0581 (18)	0.0554 (16)	0.0340 (13)	−0.0086 (14)	0.0138 (13)	0.0035 (12)
C20	0.0500 (15)	0.0402 (13)	0.0382 (13)	0.0056 (12)	0.0165 (12)	0.0045 (11)
C21	0.0407 (13)	0.0280 (11)	0.0371 (12)	0.0089 (10)	0.0116 (10)	0.0065 (9)
C22	0.0390 (12)	0.0267 (10)	0.0333 (11)	0.0026 (9)	0.0114 (10)	0.0081 (8)
C23	0.0543 (16)	0.0312 (12)	0.0383 (13)	0.0081 (11)	0.0136 (12)	0.0050 (10)
C24	0.0671 (19)	0.0366 (14)	0.0437 (14)	0.0029 (13)	0.0119 (14)	−0.0061 (11)
C25	0.0545 (18)	0.0550 (17)	0.0405 (14)	−0.0035 (14)	0.0009 (13)	−0.0001 (12)
C26	0.0418 (15)	0.0514 (15)	0.0472 (15)	0.0050 (12)	0.0026 (12)	0.0094 (12)
C27	0.0456 (14)	0.0316 (12)	0.0443 (13)	0.0068 (10)	0.0089 (11)	0.0040 (10)

### Geometric parameters (Å, °)

**Table:**

N1—C2	1.369 (3)	C15—C16	1.391 (3)
N1—C6	1.427 (2)	C15—C20	1.392 (3)
N1—C21	1.470 (3)	C16—C17	1.383 (4)
C2—O8	1.228 (2)	C16—H16	0.96 (3)
C2—N3	1.368 (3)	C17—C18	1.381 (4)
N3—C4	1.386 (3)	C17—H17	0.95 (3)
N3—H3	0.86 (2)	C18—C19	1.373 (4)
C4—O7	1.236 (2)	C18—H18	0.99 (3)
C4—C5	1.437 (3)	C19—C20	1.379 (4)
C5—C9	1.408 (3)	C19—H19	0.97 (3)
C5—C6	1.455 (3)	C20—H20	0.93 (3)
C6—N14	1.282 (3)	C21—C22	1.507 (3)
C9—N10	1.316 (3)	C21—H21A	0.97 (2)
C9—C13	1.506 (3)	C21—H21B	0.99 (2)
N10—C11	1.467 (3)	C22—C27	1.381 (3)
N10—H10	0.90 (2)	C22—C23	1.395 (3)
C11—C12	1.499 (4)	C23—C24	1.384 (4)
C11—H11A	1.01 (3)	C23—H23	0.99 (3)
C11—H11B	1.00 (3)	C24—C25	1.375 (4)
C12—C13	1.531 (3)	C24—H24	0.96 (3)
C12—H12A	1.05 (3)	C25—C26	1.379 (4)
C12—H12B	0.98 (3)	C25—H25	1.00 (3)
C13—H13A	0.97 (3)	C26—C27	1.384 (4)
C13—H13B	0.97 (3)	C26—H26	1.00 (3)
N14—C15	1.407 (3)	C27—H27	0.96 (2)
			
C2—N1—C6	122.57 (17)	C16—C15—C20	118.3 (2)
C2—N1—C21	116.70 (17)	C16—C15—N14	122.3 (2)
C6—N1—C21	118.51 (17)	C20—C15—N14	119.0 (2)
O8—C2—N3	121.31 (19)	C17—C16—C15	120.3 (3)
O8—C2—N1	122.72 (19)	C17—C16—H16	121.3 (15)
N3—C2—N1	115.97 (18)	C15—C16—H16	118.4 (15)
C2—N3—C4	126.16 (19)	C18—C17—C16	120.8 (3)
C2—N3—H3	115.4 (16)	C18—C17—H17	119.4 (18)
C4—N3—H3	118.5 (16)	C16—C17—H17	119.7 (18)
O7—C4—N3	117.81 (19)	C19—C18—C17	119.1 (3)
O7—C4—C5	126.25 (19)	C19—C18—H18	119.3 (17)
N3—C4—C5	115.94 (18)	C17—C18—H18	121.6 (17)
C9—C5—C4	118.04 (18)	C18—C19—C20	120.6 (3)
C9—C5—C6	122.50 (18)	C18—C19—H19	120.2 (18)
C4—C5—C6	119.06 (17)	C20—C19—H19	119.2 (18)
N14—C6—N1	113.57 (18)	C19—C20—C15	120.8 (3)
N14—C6—C5	131.61 (19)	C19—C20—H20	121.4 (16)
N1—C6—C5	114.81 (17)	C15—C20—H20	117.7 (16)
N10—C9—C5	124.5 (2)	N1—C21—C22	115.08 (19)
N10—C9—C13	107.55 (19)	N1—C21—H21A	107.5 (13)
C5—C9—C13	127.79 (19)	C22—C21—H21A	110.5 (13)
C9—N10—C11	114.7 (2)	N1—C21—H21B	104.6 (13)
C9—N10—H10	120.3 (16)	C22—C21—H21B	110.5 (13)
C11—N10—H10	124.8 (16)	H21A—C21—H21B	108.3 (18)
N10—C11—C12	101.68 (19)	C27—C22—C23	118.0 (2)
N10—C11—H11A	109.7 (17)	C27—C22—C21	123.7 (2)
C12—C11—H11A	115.6 (17)	C23—C22—C21	118.2 (2)
N10—C11—H11B	110.5 (16)	C24—C23—C22	120.5 (3)
C12—C11—H11B	111.4 (16)	C24—C23—H23	121.3 (16)
H11A—C11—H11B	108 (2)	C22—C23—H23	118.2 (16)
C11—C12—C13	103.9 (2)	C25—C24—C23	120.6 (3)
C11—C12—H12A	106.9 (15)	C25—C24—H24	119.8 (16)
C13—C12—H12A	108.7 (15)	C23—C24—H24	119.5 (16)
C11—C12—H12B	115.1 (15)	C24—C25—C26	119.6 (3)
C13—C12—H12B	110.6 (15)	C24—C25—H25	120.6 (16)
H12A—C12—H12B	111 (2)	C26—C25—H25	119.9 (16)
C9—C13—C12	103.0 (2)	C25—C26—C27	119.9 (3)
C9—C13—H13A	113.3 (15)	C25—C26—H26	120.6 (16)
C12—C13—H13A	117.7 (16)	C27—C26—H26	119.4 (16)
C9—C13—H13B	109.5 (18)	C22—C27—C26	121.4 (2)
C12—C13—H13B	107.9 (17)	C22—C27—H27	118.9 (14)
H13A—C13—H13B	105 (2)	C26—C27—H27	119.7 (14)
C6—N14—C15	125.54 (19)		
			
C6—N1—C2—O8	166.1 (2)	N10—C9—C13—C12	18.0 (3)
C21—N1—C2—O8	3.3 (3)	C5—C9—C13—C12	−157.6 (2)
C6—N1—C2—N3	−13.8 (3)	C11—C12—C13—C9	−28.8 (3)
C21—N1—C2—N3	−176.6 (2)	N1—C6—N14—C15	158.8 (2)
O8—C2—N3—C4	173.1 (2)	C5—C6—N14—C15	−22.4 (4)
N1—C2—N3—C4	−7.0 (3)	C6—N14—C15—C16	−42.4 (3)
C2—N3—C4—O7	−167.7 (2)	C6—N14—C15—C20	144.7 (2)
C2—N3—C4—C5	12.0 (3)	C20—C15—C16—C17	−1.0 (4)
O7—C4—C5—C9	9.8 (3)	N14—C15—C16—C17	−174.0 (2)
N3—C4—C5—C9	−169.9 (2)	C15—C16—C17—C18	0.1 (4)
O7—C4—C5—C6	−177.4 (2)	C16—C17—C18—C19	0.3 (4)
N3—C4—C5—C6	3.0 (3)	C17—C18—C19—C20	0.3 (4)
C2—N1—C6—N14	−154.0 (2)	C18—C19—C20—C15	−1.3 (4)
C21—N1—C6—N14	8.5 (3)	C16—C15—C20—C19	1.6 (3)
C2—N1—C6—C5	27.1 (3)	N14—C15—C20—C19	174.8 (2)
C21—N1—C6—C5	−170.4 (2)	C2—N1—C21—C22	84.4 (2)
C9—C5—C6—N14	−26.9 (4)	C6—N1—C21—C22	−79.1 (2)
C4—C5—C6—N14	160.5 (2)	N1—C21—C22—C27	−10.3 (3)
C9—C5—C6—N1	151.8 (2)	N1—C21—C22—C23	172.27 (19)
C4—C5—C6—N1	−20.7 (3)	C27—C22—C23—C24	−3.3 (3)
C4—C5—C9—N10	−15.1 (3)	C21—C22—C23—C24	174.3 (2)
C6—C5—C9—N10	172.4 (2)	C22—C23—C24—C25	2.5 (4)
C4—C5—C9—C13	159.9 (2)	C23—C24—C25—C26	−0.1 (4)
C6—C5—C9—C13	−12.7 (4)	C24—C25—C26—C27	−1.6 (4)
C5—C9—N10—C11	176.1 (2)	C23—C22—C27—C26	1.6 (3)
C13—C9—N10—C11	0.3 (3)	C21—C22—C27—C26	−175.8 (2)
C9—N10—C11—C12	−18.9 (3)	C25—C26—C27—C22	0.8 (4)
N10—C11—C12—C13	28.4 (3)		

### Hydrogen-bond geometry (Å, °)

**Table:**

*D*—H···*A*	*D*—H	H···*A*	*D*···*A*	*D*—H···*A*
N10—H10···O7	0.90 (2)	2.03 (2)	2.664 (3)	127 (2)
N10—H10···O7^i^	0.90 (2)	2.22 (2)	2.891 (3)	131 (2)
N3—H3···O8^ii^	0.86 (2)	2.10 (2)	2.937 (3)	165 (2)

Symmetry codes:  (i) −*x*+1, −*y*+1, −*z*+1; (ii) −*x*, −*y*, −*z*+1.
